# Close-to-community providers of health care: increasing evidence of how to bridge community and health systems

**DOI:** 10.1186/s12960-016-0132-9

**Published:** 2016-06-02

**Authors:** S. Theobald, K. Hawkins, M. Kok, S. Rashid, D. G. Datiko, M. Taegtmeyer

**Affiliations:** Liverpool School of Tropical Medicine, Pembroke Place, Liverpool, L3 5QA United Kingdom; Pamoja Communications, Brighton, United Kingdom; Royal Tropical Institute, Amsterdam, Netherlands; James P Grant School of Public Health (JPGSPH), BRAC University, Dhaka, Bangladesh; HHA - REACH Ethiopia, Hawassa, Ethiopia

## Introduction

The recent thematic series on close-to-community providers published in this journal brings together 14 papers from a variety of contexts and that use a range of research methods. The series clearly illustrates the renewed emphasis and excitement about the potential of close-to-community (CTC) providers in realising universal health coverage and supporting the sustainable development goals. This editorial discusses key themes that have emerged from this rich and varied set of papers and reflect on the implications for evidence-based programming. We are at a critical stage in the development of CTC programming and policy which requires the creation and communication of new knowledge to ensure the safety, sustainability, quality and accessibility of services, and their links with both the broader health system and the communities that CTCs serve.

## Contexts and methods

The series has a focus on low- and middle-income countries where the coverage of the health system is suboptimal, inequities may be stark and community health programmes are being scaled up. The papers include the analysis of empirical research in a number of African (Democratic Republic of Congo, Ghana, Senegal, Uganda, Zimbabwe, Tanzania, Ethiopia and Mozambique) and Asian (Bangladesh, India) contexts as well as international analysis from the literature and different data sets.

The methods deployed are largely qualitative capturing the depth and detail of context and the voice and experience of CTC providers and other stakeholders. Qualitative methods deployed include key-informant, in-depth and semi-structured interviews and focus group discussions with CTC providers, their supervisors, managers and community members. These are often complemented by document review (for example, [1–6]). In their work on Uganda, Turinawe et al. [7] also use qualitative research from an ethnographic perspective, complementing their insights with participant observation. Three papers take a mixed-method approach blending analysis from questionnaires involving CTC providers and qualitative research with CTC providers and other stakeholders [8], combining qualitative research with two quantitative measurements: (1) a survey of health worker satisfaction and motivation and (2) a clinical knowledge assessment focusing on maternal, newborn and child health [9] or through supporting qualitative case studies on the implementation of community health system strengthening (CHSS) approaches with health facility data [10]. Mpembeni et al. [11] present findings from a survey using questions on the Likert scale and factor analysis.

Other methods include the following: an exploratory review on the costs and cost-effectiveness of community health workers (CHWs), which drew on a larger systematic review on effectiveness [12]; the development of a strategic community health system partnership through a yearlong evidence synthesis exercise on CHW performance, synthesis records, author consultations and expert review [13]; and reflections on a photo competition on gender and health systems, with many entries featuring CTC providers, including the ensuing judging process and email dialogue with participants who reacted to the photo competition [14].

The qualitative papers enable the reader to understand CTC providers as women and men who are negotiating between communities and health systems and often acting as a bridge between them in various contexts. To date, CTC provider voices, experiences and perspectives have been neglected in health system strengthening and research. These voices need to be heard: the rich qualitative insights presented in this supplement show how CTC providers exert agency and make choices in challenging scenarios. In addition, patient and broader community perspectives are frequently missing in literature and policy documents. In the review on cost and cost-effectiveness conducted by Vaughan et al. [12], most studies took a programme cost perspective, omitting societal and patients’ perspectives and failing to capture societal costs and benefits. Non-tangible costs and non-health benefits commonly associated with CHWs, such as increased trust between the community and health sector and strengthened relationships were not taken into account (probably because these are hard to quantify and cost). Patient costs were considered in only 11 of the 36 studies included in the review. Vaughan et al. [12] argue that a mixed-method approach to costing and cost-effectiveness studies that include societal costs would add the much-needed depth to future costing or cost-effectiveness studies.

## CTC providers: understanding heterogeneity

CTC providers are known by different names and titles in different contexts. These include the following: community health workers, community distributors, community health nurses, community-directed health workers, health auxiliaries, health promoters, family welfare educators, health volunteers, village health workers/volunteers/teams, community health aides, barefoot doctors, traditional healers, practitioners who combine traditional and modern medicine, allopathic practitioners, drug sellers and faith healers.

The contexts and range of CTC providers covered in this supplement are incredibly diverse. Across Ethiopia, female health extension workers (HEWs) have been selected and recruited by the government with involvement of the community, work on 16 health packages and are salaried members of the health system working within a structured process of community participation. As Kok et al. [4] write, HEWs are supported by the Health Development Army (HDA) consisting of “model families” who become leaders of a group of five families known as the “one-to-five network”, who in turn form a “development group” of 25 to 30 households within a village. This contrasts with urban Bangladesh where there is a complex array of plural formal and informal providers. Mahmud et al.’s [5] analysis draws on research which shows that the estimated density of informal CTC health service providers was 127.2 per 10 000 population in Bangladesh in 2007, 12 times higher than that of the formal CTC health service providers. Mahmud et al. [5] argue that informal CTC providers are more likely to be accessed by poor urban women and men and that developing strategic partnerships between formal and informal CTC providers will ensure better referral between the sectors and improve the quality of care.

## Strategies to support CTC providers’ interface role between communities and the health system

Despite different starting points and methods, the papers by Kok et al., Naimoli et al. and Raven et al. [1, 4, 13] reach similar conclusions: we need robust, transparent and responsive strategies to support CTC providers to best realise their vital interface role between communities and health systems. Naimoli et al. [13] argue that CHWs function at the intersection of two dynamic, overlapping systems—the health system and the community—and that the support from these systems is not necessarily appropriate, strategic, collaborative or coordinated. Based on a yearlong synthesis process and expert engagement, they argue that four strategies cement support from health systems *and* communities: (1) joint ownership and design of CHW programmes, (2) collaborative supervision and constructive feedback, (3) a balanced package of incentives and (4) a practical monitoring system incorporating data from communities and the health system. The comprehensive implementation of those four strategies has the potential to yield great gains for CHW performance at scale.

Drawing on evidence from the Democratic Republic of Congo, Ghana, Senegal, Uganda and Zimbabwe, Raven et al. [1] also argue for the importance of transparent and accountable strategies within health systems and communities to support CTC providers. They state that CTC providers, like all health workers, require a holistic and transparent set of human resource management strategies and processes. For CTC providers, whose relationships with the health system may be more tenuous than nurses or doctors, for example, human resource management is all the more critical as a way to cement supportive relationships between CTC providers, health systems and communities. Raven et al. [1] explore issues of attraction and retention of CHWs, their recruitment and selection and the performance management of CHWs once engaged. They argue that many human resource management practices are in place, but how well they are implemented, the degree to which they meet the expectations of the CHWs and their effects on human resource outcomes vary across contexts. They call for a stronger human resource management approach with coordinated action across three groups of management actors (frontline supervisors, programme managers and community members). Many of the empirical papers explore some of these strategies in more detail, and these are discussed next.

## The role of the community in the selection and support of CTC providers

In Raven et al.’s [1] qualitative research, respondents described several problems related to CHW selection. These included the following: nepotism in the selection process, that willing applicants may not meet selection criteria (for example, older women and men may not meet the educational criteria), the difficulty in recruiting young literate CHWs, early dropout due to misinformation about the job and too few people volunteering. Naimoli et al. [13] discuss the importance of community participation in joint ownership and design of CHW programmes, of which selection is a component.

Turinawe et al. [7] used an ethnographic approach where they retrospectively analysed the process of selecting Village Health Teams (VHTs) through a process supported by AMREF in rural Uganda. Their research describes how a poorly executed recruitment process for VHTs, which was over-influenced by community elites such as village council leaders, negatively impacted upon CTC providers’ ability to work and the respect and trust of communities towards those who were selected. Elite capture was observed: local leaders selected VHTs in a way that alienated community members who in turn started to question the credentials of those who were selected. VHTs felt under pressure to regulate community members and to report and “arrest” those who, for example, did not have a toilet. Resentment grew and, without the support of the community, the VHTs had low morale and stopped work.

Similarly, in rural Manipur, India, qualitative research by Saprii et al. [6] showed that the selection of the role of Accredited Social Health Activists (ASHAs) was often skewed by political interests. Most doctors and nurses interviewed felt that there was “biased” selection of ASHAs. Although ASHAs were nominated by the village community, the final selection was viewed as based on favouritism and unduly influenced by local leaders who hoped to one day become permanent government employees. This in turn damaged the legitimacy and ability of ASHAs to continue their work.

More positive examples of engaging communities to support CTC providers emerged from the analysis of a community health system strengthening (CHSS) approach in Uganda and Tanzania by Lunsford et al. [10]. CHSS draws on existing formal and informal networks within a community, such as agricultural or women’s groups, to support CTC providers and address gaps in community-based health services. Community team members supported the CTC providers, encouraging community members to follow advice and referrals from CTC providers, thereby reducing loss to follow-up at all points in the continuum of care. The results were encouraging: during implementation, more pregnant women registered for antenatal care and tested for HIV, health extension workers conducted more postnatal visits and more households had functioning latrines and proper latrine use increased. The model offers a framework for bringing representatives from existing community networks, CTC providers and health facility staff together to form a community team charged with identifying challenges in service delivery, testing solutions and monitoring changes.

The CHSS approach has synergies with the natural helper model proposed by Turinawe et al. [14] as a way to avoid elite capture. In this model, naturally existing informal helping networks, including volunteers already trusted by the people being served, are used to inform CTC provider selection and support the CTC providers in conducting their tasks, resulting in positive and supportive relationships.

## The need to move to supportive, structured relationships in CTC supervision

Naimoli et al. [13] argue that the supervision of CHWs has been a persistent weakness in large-scale, national programmes and that accountability to communities has often been lacking. This analysis is echoed by Raven et al. [1], who argue for the importance of collaborative supervision between programme managers, supervisors and communities. The concept of supportive supervision as an interactive process is gaining traction (and is a key element of quality improvement cycles within CTC services). However, it is not without challenges. In rural Ghana, Sacks et al. [9] reported that community health nurses generally reported good relationships with colleagues and that they were respected by patients. However, they desired more respect from supervisors and also wanted more training—especially those posted at the community level as compared to those at health facilities.

Qualitative research on supervision in Mozambique by Ndima et al. [3] demonstrated that the supervision of Agentes Polivalentes Elementares (APEs) should have been structured according to policy; however, in reality, it had a number of flaws. Supervision was irregular and infrequent, focused more on fault-finding than being supportive and did not address all areas of the APE’s work, which APEs found demotivating. Supervisors felt unsupported and under-resourced and this had negative repercussions on their performance. In this context, community governance and accountability mechanisms were only partially able to fill the gaps left by the supervision provided by the health system. Ndima et al. [3] argue for an improved supportive and group-led supervision system to enhance the APE’s motivation and performance.

Similarly, Kok et al.’s [4] qualitative analysis in Ethiopia found that supervision was top-down and often more fault finding than supportive. The authors also recommend exploring peer-based supportive supervisory processes as well as ensuring planned and communicated supervision, as health centre staff can be overloaded with other work.

Roberton et al. [8] report on an integrated maternal, newborn and child health (MNCH) programme in Tanzania which has made progress towards meeting its objective of supportive, community-embedded supervision through a process of cascade training of facility health workers in conducting supportive supervision of voluntary CHWs. Their multi-method evaluation revealed that CHWs appreciate the sense of legitimacy that arises when supervisors visit them in their village and valued the supervision process. However, supervision was still relatively infrequent (once every 2.8 months, as opposed to the ideal of once a month) and CHWs and supervisors still saw supervision primarily as an opportunity to check reports, rather than to mentor, support and solve problems. The supervisors, health workers at the health facility, were removed from the working realities of CHWs. Roberton et al. [8] discuss whether health workers are best placed to take on this critical supervisory role or whether involving other community members may be more strategic, especially with respect to advising on CHWs’ community engagement role. The authors argue that supportive and engaging supervision needs to be informed by both health systems and community perspectives.

## The critical role of programme design, motivation and incentives in responsive and people-centred health systems

Naimoli et al. [13] stress the importance of a balanced package of incentives for CTC providers. Raven et al. [1] discuss the differences between incentivising volunteers and paid CTC providers, arguing that intrinsic motivation is likely to be particularly important for volunteers. Mpembeni et al.’s [11] quantitative research in Tanzania, in the context of a MNCH programme, revealed that people were more motivated to become CHWs due to altruism (work on MNCH, desire to serve God, work hard) and intrinsic needs (help community, improve health, pride) than due to external stimuli (monetary incentives, skill utilisation, community respect or hope for employment). The factor analysis showed that CHWs were satisfied by relationships with health workers and communities, job aids and the capacity to provide services but were dissatisfied with the lack of transportation, communication devices and financial incentives for carrying out their tasks. The strongest satisfaction factor for CHWs was related to work relations with varied CHW stakeholders—including the community—and training. CHWs’ expectations regarding incentives can change over time: although CHWs joined mainly due to their altruistic nature, they became discontented with the lack of monetary compensation, transportation and communication support received. In rural Ghana, more than 60% of CHNs indicated that they were not satisfied with the pay and did not believe that they were being fairly compensated; staff shortages, insufficient space and demand for further training also emerged as key issues [9].

The importance of incentives and their impact on performance is demonstrated by Saprii et al.’s [6] qualitative analysis of the role of ASHAs in rural India, which reveals that small and irregular monetary incentives demotivate ASHAs and also skew priorities and action. Here, both performance-based incentives and the nature of supportive supervision encouraged ASHAs to focus on promoting bio-medical care (in particular, institutional delivery) and in achieving targets set by the health facility, while community mobilisation, a core yet non-incentivised aspect of ASHAs’ roles, got neglected.

Qualitative research in Mozambique by Give et al. [2] also reveals how demand and supply factors interplay to shape the APE’s task prioritisation and hence impact on health equity. Give et al. [2] argue that APEs found themselves caught between community demands for broader curative services while the official policy largely limits their focus. APEs showed agency in co-constructing a middle path acceptable to all. Saprii et al., Kok et al. and Give et al. [2, 4, 6] demonstrate the impact of weak health systems on CTC provider credibility and ability to fulfil their role. For example, in India, the health centres that ASHAs link to are ill-equipped and poorly functioning with negative consequences for the ASHA’s ability to inspire trust and credibility in the community [6]. CTC providers’ legitimacy is arguably linked to the strengths of the health system, and as argued by many in this series, strengthening CTC providers’ ability to promote universal health coverage and quality care requires the simultaneous strengthening of health systems.

## Negotiating trusting relationships

Trusting relationships, and how they are negotiated and experienced, emerged as a key theme throughout many of the papers. Supportive supervision, community engagement in selection, support of CTC providers and fair and transparent approaches to the provision of incentives can all help nurture trusting relationships. Lunsford et al. [10] argue that in Tanzania and Ethiopia a valuable trust relationship was created between team members and the CTC providers which supported positive outcomes. While Kok et al. [4] argue that in Ethiopia trust, communication, dialogue and expectations influenced the strength of relationships between CTC providers and the communities and the health system structures they link to. Where HEWs were selected by their communities, trust and engagement between them was enhanced. However, from the health sector side, top-down supervision and inadequate training possibilities hampered trusting relationships and demotivated HEWs.

In the qualitative research on menstrual regulation in Bangladesh, in all four research sites, informal providers were better able than formal providers to gain the trust of the community and build positive relationships. This was due to their cultural and social familiarity and a “comfort zone” which meant that clients were able to interact with providers discreetly regarding their maternal, sexual and reproductive health (MSRH) problems. Mahmud et al. [5] argue that key factors influencing community choice of provider are availability, accessibility, expenses (clients need to pay the formal CTC providers in cash, while for the informal CTC health service providers, remuneration is not always monetary and can also include in-kind payments and payments on credit) and perceived quality of care, which is shaped by notions of trust, respect and familiarity. Informal providers are usually the first point of contact even for those clients who subsequently access MSRH services from formal providers. The authors argue for the importance of enhancing trusting partnerships between formal CTC providers and more informal providers as strategies to enhance women’s access to quality MSRH care that respects and responds to their needs and concerns. In Bangladesh, despite existing informal interactions between both types of providers and a shared understanding that this can be beneficial for clients, there is no effective link or partnership between these providers for referral, coordination and communication.

Strategies to enhance trust between the multiple players involved in CTC service provision will have positive dividends. However, as Naimoli et al. [13] caution, trusting relationships are not born, they must be made, and they require clear transparent approaches to governance, willingness, a common plan and flexibility. Careful planning and understanding of the motivation and incentives for all involved will also be important.

## Power relationships and gender roles shape CTC interactions at multiple levels

Power and the way it is mediated by gender, age and other axes of inequality play out in numerous ways and at different levels. Gender roles, relations and norms in different countries also effect whether women or men are chosen to become a CTC provider. Naimoli et al. [13] state that in many countries, women figure prominently as CHWs, and George et al. [14] through their photo analysis argue that “women on the frontline of health service delivery” was a key theme portrayed by 17 photos. This varies by context; in Ethiopia, by policy, all HEWs and HDA leaders are women and this was found to be positively valued by the community, because of the perceived cultural suitability of women handling reproductive health issues. Raven et al.’s [1] analysis shows that in the Democratic Republic of Congo (DRC), Senegal, Uganda and Zimbabwe, CHWs were reportedly more likely be female and aged over 30 years as they were seen as being more interested in health issues, are already involved in health within families, are respected and listened to within communities and are perceived as being able to work easily with people. Older women were reported to be more likely to become CHWs as they have experience of looking after children while younger, unmarried women are seeking salaried employment. However, in Ghana, CHWs were more likely to be male as women are more occupied with taking care of their farms, homes and families.

George et al. [14] argue that women’s role as unpaid carers for family members emerged as a theme in their photo analysis. Caring for sick or elderly family members is often not recognised as work by the health sector. Yet the visible face of frontline health and community systems is often female. Whether paid or unpaid, CTC providers can face blurred boundaries in their role, they do not go into the office or knock off at five and in reality they can be on call to respond to community needs around the clock. In many contexts, women also have the responsibility for domestic, reproductive and caring roles, so managing the expectation of being a CTC provider makes this additionally challenging. Women’s labour is too often equated with volunteerism, and CTC providers are arguably more likely to be female if these are unpaid volunteer roles. As Naimoli et al. [13] argue, there is need to better understand the dynamics of gendered recognition, mobility and reimbursement as these strategies will influence women’s overall sense of autonomy and agency and affect the power dynamics within families and beyond. No papers analysed how gender shapes CTC providers’ room for manoeuvre to interact with and shape future health systems.

## Conclusion

There is a need for evidence to inform the scaleup of CTC programmes which is pragmatic and contextually embedded and also focuses on the quality of the services provided. We need to deploy a range of methods. As demonstrated in this series, qualitative and mixed methods are valuable as these allow for the findings to be embedded in the context of the programme and typology of the CTC providers and enable an assessment of what works, why and for whom. This series demonstrates that when it comes to CTC programmes there is little possibility of a one-size-fits-all blueprint approach given the realities of the inter-relationships between communities and the health system and the variable implementation of formal policies across the board.

In recent years, there has been a welcome push for “people-centred health systems”. Many of the articles in the collection suggest that when it comes to CTC providers we must pay attention to the “human” in human resource management. CTC providers are individuals and are negotiating and living a challenging interface as the bridge between communities and health systems. A CTC health programme scaleup is challenging in the face of health system constraints, and there is a clear need for strategies and approaches to support both health system strengthening and community engagement. There is need for innovation in multiple levels. Strategies to support embedment of CTC services so that districts, CTC provider and communities can positively partner to deliver quality services will be important as will innovation with new technologies: mobiles and smart phones in the hands of CTC providers have the potential to democratise information and data sharing. We predict that the debates on the best ways to support and utilise CTCs will continue. We need further evidence and experience sharing to support CTCs to be fit for purpose in different contexts to promote universal coverage and support the achievement of the Sustainable Development Goals.
